# Predator-induced defences in *Daphnia pulex*: Selection and evaluation of internal reference genes for gene expression studies with real-time PCR

**DOI:** 10.1186/1471-2199-11-50

**Published:** 2010-06-29

**Authors:** Katina I Spanier, Florian Leese, Christoph Mayer, John K Colbourne, Don Gilbert, Michael E Pfrender, Ralph Tollrian

**Affiliations:** 1 Ruhr-University Bochum, Department of Animal Ecology, Evolution and Biodiversity, D-44780 Bochum, Germany; 2 University of Notre Dame, Department of Biological Sciences, Notre Dame, IN 46556 USA; 3 Indiana University, The Center for Genomics and Bioinformatics, Bloomington, IN 47405 USA

## Abstract

**Background:**

The planktonic microcrustacean *Daphnia pulex *is among the best-studied animals in ecological, toxicological and evolutionary research. One aspect that has sustained interest in the study system is the ability of *D. pulex *to develop inducible defence structures when exposed to predators, such as the phantom midge larvae *Chaoborus*. The available draft genome sequence for *D. pulex *is accelerating research to identify genes that confer plastic phenotypes that are regularly cued by environmental stimuli. Yet for quantifying gene expression levels, no experimentally validated set of internal control genes exists for the accurate normalization of qRT-PCR data.

**Results:**

In this study, we tested six candidate reference genes for normalizing transcription levels of *D. pulex *genes; alpha tubulin (aTub), glyceraldehyde-3-phosphate dehydrogenase (GAPDH), TATA box binding protein (Tbp) syntaxin 16 (Stx16), X-box binding protein 1 (Xbp1) and CAPON, a protein associated with the neuronal nitric oxide synthase, were selected on the basis of an earlier study and from microarray studies. One additional gene, a matrix metalloproteinase (MMP), was tested to validate its transcriptional response to *Chaoborus*, which was earlier observed in a microarray study. The transcription profiles of these seven genes were assessed by qRT-PCR from RNA of juvenile *D. pulex *that showed induced defences in comparison to untreated control animals. We tested the individual suitability of genes for expression normalization using the programs geNorm, NormFinder and BestKeeper. Intriguingly, Xbp1, Tbp, CAPON and Stx16 were selected as ideal reference genes. Analyses on the relative expression level using the software REST showed that both classical housekeeping candidate genes (aTub and GAPDH) were significantly downregulated, whereas the MMP gene was shown to be significantly upregulated, as predicted. aTub is a particularly ill suited reference gene because five copies are found in the *D. pulex *genome sequence. When applying aTub for expression normalization Xbp1 and Tbp are falsely reported as significantly upregulated.

**Conclusions:**

Our results suggest that the genes Xbp1, Tbp, CAPON and Stx16 are suitable reference genes for accurate normalization in qRT-PCR studies using *Chaoborus*-induced *D. pulex *specimens. Furthermore, our study underscores the importance of verifying the expression stability of putative reference genes for normalization of expression levels.

## Background

Gene-expression studies provide insights into the regulatory processes of genes that modulate phenotypes of organisms. The two most reliable techniques to date that directly measure and compare the differential response in gene expression are microarray studies and quantitative real-time reverse transcription polymerase chain reaction (qRT-PCR) analyses. Microarrays provide a high-throughput measurement of the transcriptional changes for thousands of genes, within short time, from limited number of RNA samples. However, quality of expression data can vary substantially and is often validated by a second method. In contrast to microarrays, qRT-PCR provides precise quantification over a wider dynamic range. Because of the higher sensitivity, qRT-PCR is often used to validate microarray data. Several variables can still introduce biases in qRT-PCR studies. For example, the amount and quality of template RNA, the enzymatic efficiencies and other *in vitro *artefacts can add technical variability to the data y[1-4]. Hence, experiments are typically standardized and expression levels need to be normalized [5,6]. Normalization is accomplished in several ways; the expression values from target genes of interest can be balanced against the total amount of RNA in the reactions, balanced against synthetic RNA that is "spiked" in each reaction, or measured against internal reference genes. This last method is generally considered reliable and is frequently applied for quantifying relative gene expression [7]. Yet, several studies have shown that this approach can introduce large errors when the expression of such "housekeeping genes" varies under different treatments and in different tissues [e.g. [8]]. To improve robustness of the experiment, it is recommended to use more than one reference gene [7,9] and to verify that their transcriptional activity are stable across conditions and tissue types. Because it is difficult to assess the expression stability of a reference gene by itself, current approaches aim to analyze the expression levels of several candidate reference genes with respect to each other. In samples with different amounts of input RNA the ratio of two ideal reference genes remains constant.

Once suitable reference genes for a certain experiment are selected, a normalization factor (NF), which is the geometric mean of the crossing point (CP) values of the reference genes, is calculated for normalization of the genes under investigation, i.e. to remove nonspecific variation in the data.

### Selection of reference genes for *Daphnia pulex*

So far, no systematic validation of reference genes in *D. pulex *has been published. In a recent study, Schwarzenberger et al. [10] tested the expression stability of seven genes in a different species, *D. magna*, and found glyceraldehyde-3-phosphate dehydrogenase (GAPDH), TATA box binding protein (Tbp), and succinate dehydrogenase (sucDH) suitable for normalization of gene expression in predator experiments (fish and phantom midge larvae *Chaoborus*). Under low-food quality conditions (microcystin-producing strain of the cyanobacterium *Microcystis*), Tbp, 18S, and alpha tubulin (aTub) were suitable reference genes and GAPDH and ubiquitin conjugating enzyme (UBC) significantly upregulated [10]. Heckmann et al. [11] investigated the expression stability of several genes also in *D. magna*, when exposed to ibuprofen. They selected GAPDH, an actin gene (similar to actin isoform 3 in *D. pulex*) and UBC as the most stable reference genes and showed that aTub was differentially regulated. Rider and LeBlanc [12] and Zeis et al. [13] used beta-actin as a single reference gene for *D. magna *without prior validation of its suitability for expression normalization. The candidate reference genes chosen for our analysis include three frequently applied housekeeping genes, aTub, GAPDH, Tbp, and three genes with microarray support for stable expression: Syntaxin 16 (Stx16), X-box binding protein 1 (Xbp1) and CAPON (see Table 1). Xbp1 is a transcription factor which is activated through differential splicing. It plays a major role in unfolded protein response in eukaryotes [14] and is critical for larval development of *Drosophila *[15].

**Table 1:** Candidate reference and differentially expressed genes with putative function and gene ID from the Dappu V1.1 draft genome annotation, primer sequences, amplicon characteristics.

Gene symbol	Gene name	(putative) Function	Gene ID	P	Primer sequences [5'→3']	L (bp)	Localization in gene	E (%)
aTub	alpha Tubulin	Cytoskeletal protein	Dappu-301837	5	GCATGTTGTCCAACACTACTGC	135	3' exon	91
					CCTCAGAGAACTCTCCCTCCTC			
GAPDH	Glyceraldehyde-3-phosphate dehydrogenase	Glycolytic enzyme	Dappu-302823	0	TGGGATGAGTCACTGGCATAC	136	3' exon	93
					GAAAGGACGACCAACAACAAAC			
Tbp	TATA binding protein	Transcription initiation	Dappu-194512	0	CTACGATGCATTCGATAACATATACC	144	3' exon	90
					AGAACCAGCAATGAGTTAAACAAAG			
Stx16	Syntaxin 16	Protein involved in exocytosis	Dappu-194044	0	CACATTGGTCGTCCTTAGTCTTG	148	3' exon	93
					TGCTATACGTTACGCTTGTCCTTAC			
Xbp1	X-box binding protein 1	Transcription factor	Dappu-314438	0	CCGATATTCGAGACTGCAATG	131	3' exon	93
					AAAGATGGGTGAGCCAGAAATAC			
MMP	Matrix metallo-proteinase	Degradation of extracellular proteins	Dappu-303491	0	CGAAACATGGACGCATAACTC	80	spanning penultimate 3' intron	92
					GTCCCAAAGTGTGACCGAAC			
CAPON	C-terminal pdz ligand of neuronal nitric oxide synthase	Location of neuronal nitric oxide synthase	Dappu-100564	0	TAACGAGTCGGGAGGAAGTG	140	3' exon	94
					GCTGGACTTGAGCCAGTATCTC			

Abbreviations: E: amplification efficiency; P: number of paralogs in *D. pulex *genome; L: length of the amplicon

CAPON is a protein which targets the neuronal nitric-oxide synthase to the presynaptic nerve terminal in mice [16]. To test the performance of candidate reference genes in an actual experiment, one gene with expected differential expression levels encoding for a matrix metalloproteinase (MMP) was selected on the basis microarray data (manuscript in preparation). MMPs are a family of evolutionary conserved extracellular proteases that play important roles in cell-cell signaling processes in most animal species [17-19]. Most importantly, they process and degrade extra- and pericellular proteins. A precise function of MMP in *D. pulex *has not yet been described.

The expression levels of these seven genes were measured by qRT-PCR using *D. pulex *juveniles that were exposed to water-borne chemical cues (kairomones) released by *Chaoborus *larvae and manifested the characteristic defence-against-predator phenotype called neckteeth ('induced' animals) [20]. These recorded gene transcript levels were compared to expression levels in unexposed juveniles without neckteeth (control) that have not been exposed to *Chaoborus *larvae. To date, no single best strategy for the selection of reference genes exists. Therefore, the suitability of the genes as reference markers for normalization was assessed using three different algorithms implemented in the programs BestKeeper [9], geNorm [7] and NormFinder [21] and evaluated by normalizing the expression level of a regulated gene against different sets of the candidate reference genes.

## Results and discussion

### RNA Quality

RNA concentration and purity was measured with the NanoDrop ND-1000 spectrophotometer (NanoDrop Technologies). The mean (± SD) A260/280 ratio of the samples was 2.02 ± 0.05, indicating pure (protein free) RNA quality. RNA integrity of samples was further checked by capillary gel electrophoresis on the StdSens chip of the Experion RNA StdSens Analysis Kit (Bio-Rad).

### qRT-PCR efficiencies and intra-assay variation

The expression levels of the seven candidate reference genes were measured in triplicates with qRT-PCR in 12 biological replicates of each induced and non-induced daphnids (50 individuals per replicate). The CP values, which negatively correlate with the concentration of target sequence present at the very beginning of the amplification reaction [22], ranged from 19.72 cycles for the gene with the highest expression (GAPDH) to 28.95 cycles for MMP, which showed the lowest expression (Table 2). The standard deviation (SD) within triplicates ranged from 0.044 to 0.532 cycles with a mean of 0.242 cycles. Mean PCR efficiencies varied from 90% to 94% (Table 1).

**Table 2:** Descriptive statistic analysis with BestKeeper

Gene	aTub	GAPDH	Tbp	Stx16	Xbp1	MMP	CAPON
N	24	24	24	24	24	24	24
GM [CP]	20.83	19.7	27.66	26.88	26.41	28.89	27.73
AM [CP]	20.87	19.72	27.68	26.9	26.44	28.95	27.74
Min [CP]	19.19	18.67	26.74	25.49	24.81	24.63	26.43
Max [CP]	26.84	23.58	30.16	30.71	29.14	33.67	30.21
SD [CP]	0.82	0.65	0.65	0.77	0.86	1.56	0.71
CV [% CP]	3.92	3.29	2.37	2.86	3.24	5.37	2.54
Min [x-fold]	-2.68	-1.89	-1.73	-2.36	-2.69	-13.75	-2.26
Max [x-fold]	36.98	10.96	4.4	10.6	5.43	18.92	4.72
SD [± x-fold]	1.62	1.47	1.47	1.58	1.66	2.51	1.52

BK Corr [r]	0.875	0.84	0.952	0.952	0.978	0.751	0.884
p-value	0.001	0.001	0.001	0.001	0.001	0.001	0.001

BK Corr -MMP [r]	0.943	0.917	0.922	0.982	0.944		0.803
p-value	0.001	0.001	0.001	0.001	0.001	0.001	0.001

Abbreviations: N: number of samples; GM [CP]: the geometric mean of CP; AM [CP]: the arithmetic mean of CP; Min [CP] and Max [CP]: the extreme values of CP; SD [CP]: the standard deviation of the CP; CV [% CP]: the coefficient of variance expressed as a percentage on the CP level; Min [x-fold] and Max [x-fold]: the extreme values of expression levels expressed as an absolute x-fold over- or under-regulation coefficient; SD [± x-fold]: standard deviation of the absolute regulation coefficients; BK CorrC [r]: Pearson correlation coefficient, correlation between the *BestKeeper *index and the contributing gene. BK Corr -MMP [r]: Pearson correlation coefficient between BestKeeper index without MMP in the analysis and the contributing gene.

### Descriptive analysis of the reference genes

The expression variation of each candidate reference gene was assessed using BestKeeper v. 1.0 [9]. Pfaffl et al. [9] recommend to exclude genes with a SD of the mean CP of > 1 from the NF (here called BestKeeper Index), which corresponds to a starting template variation by the factor two. MMP had a SD [CP] of 1.56 and was therefore excluded for further analyses (Table 2). The remaining genes showed minor fluctuations in expression levels (0.65 < SD [CP] < 0.86) and a strong correlation with the BestKeeper index (coefficient of correlation r between 0.803 and 0.982) after the exclusion of MMP, which indicates expression stability. The expression of all genes highly correlated with the NF, which is supported by p-values < 0.001.

### Ranking the candidate reference genes

**Figure 1:**
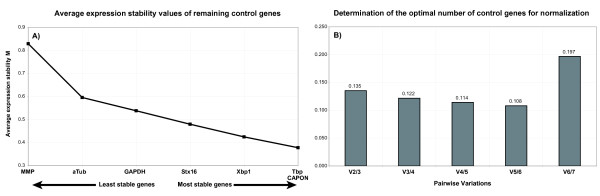
**Stability of the investigated candidate reference genes (A) and pairwise variations (B) calculated with geNorm**.

The candidate reference genes were ranked with respect to their suitability as reference genes using the programs geNorm v. 3.5 [7] and NormFinder v.0.953 [21]. geNorm utilizes a pairwise comparison approach and calculates a gene-stability measure M, which is the arithmetic mean of the pairwise variations between a particular gene and all other candidate control genes. The least stable genes have the highest M values and are successively excluded. The program also indicates the minimum number of reference genes that should be included in the NF by calculating the pairwise variation V reflecting the effect of the inclusion of an additional control gene on the NF. The authors of geNorm suggest the analysis of at least 8 samples per group and 5 to 10 candidate genes. Figure 1A shows the stepwise exclusion of the least stable genes and the average expression stability measure M of the remaining genes. The last two genes could not be further ranked because the calculation involves the ratio of expression levels. Also in this calculation, MMP was the first gene to be excluded because of the high value for M. The results of geNorm suggest Tbp and CAPON as the most stably expressed reference genes. However, Vandesompele et al. [7] highly recommended using at least three reference genes and a cut-off value of pairwise variation of 0.15. Starting with Tbp and CAPON we included Xbp1 as a third reference gene (Figure 1B). The pairwise variation for the inclusion of Xbp1 was 0.135 (V2/3) and thus lies below the suggested cut-off. Pairwise variation even further decreased with the inclusion of Stx16 (V3/4: 0.122), GAPDH (V4/5: 0.114) and aTub (V5/6: 0.108) and exceeds the 0.15 pairwise variation cut-off only after the inclusion of MMP (V6/7: 0.197).

The second program utilized was NormFinder. It applies a model-based approach, which in contrast to geNorm allows the assignment of groups to the samples (treatment vs. control). Inter- and intragroup variations are used for the calculation of a stability value i.e., candidates with minimal combined intra- and intergroup variation are ranked as the most stable genes. This approach has advantages over the pairwise comparison approach of geNorm if coregulated genes, inappropriate as reference genes, could bias the results [21].

In a first analysis with NormFinder, all seven genes were tested for intra- and intergroup variation by assigning the samples to the two groups (induced and control).

The intergroup variation was very high for MMP (± 0.476) and lowest for those of CAPON, Tbp and Xbp1, which lay between ± 0.008 and ± 0.037 (Table 3). The program indicated Xbp1 as the best reference gene with a stability value of 0.079 and Tbp and Xbp1 as the best combination of two genes with an even better combined stability value of 0.075.

**Table 3:** Candidate reference gene inter- and intragroup variation (conrol vs. induced specimens).

Gene name	Stability value	Intergroup variation		Intragroup variation	
		Induced	Control	Induced	Control
Xbp1	0.079	0.037	-0.037	0.02	0.005
Tbp	0.092	0.019	-0.019	0.06	0.014
CAPON	0.101	0.008	-0.008	0.163	0.004
Stx16	0.175	-0.121	0.121	0.062	0.001
aTub	0.337	-0.202	0.202	0.409	0.046
GAPDH	0.338	-0.217	0.217	0.128	0.124
MMP	0.602	0.476	-0.476	0.742	0.468

**Best gene**				**Xbp1**	
**Stability value**				**0.079**	
**Best combination of two genes**				**Tbp and Xbp1**	
**Stability value for best combination of two genes**				**0.075**	

Ranking according to expression stability calculated with NormFinder (NormFinder I analysis)

The NormFinder approach attempts to compensate for expression differences between treatment and control by selecting combinations of genes with opposite expression and as little intra- and intergroup variation as possible. Therefore, in our second NormFinder analysis, those genes with a high intergroup variation (aTub, GAPDH and MMP) - and thus a high bias on the selection of the best genes for normalization - were excluded (Table 4, NormFinder II analysis). The analyses of these genes in this study revealed that Stx16 has the lowest, i.e. best stability value in combination with Xbp1 (0.076), although Stx16 alone had the highest, i.e. worst stability value (0.146). The variations in expression levels are opposite and thus compensate for each other.

**Table 4:** NormFinder analysis of the four genes with the lowest (best) stability value in the previous analysis (NormFinder II analysis)

		Intergroup variation		Intragroup variation	
	Stability value	Induced	Control	Induced	Control
Xbp1	0.088	0.051	-0.051	0.024	0.006
Tbp	0.093	0.033	-0.033	0.079	0.013
CAPON	0.102	0.022	-0.022	0.02	0.044
Stx16	0.146	-0.107	0.107	0.288	0.04

**Best gene**				**Xbp1**	
**Stability value**				**0.088**	
**Best combination of two genes**				**Xbp1 and Stx16**	
**Stability value for best combination of two genes**				**0.076**	

The three different algorithmic approaches applied in this study yielded mostly concordant results. All programs clearly identified MMP as an unstably expressed gene. BestKeeper, however, did not provide information, which of the remaining genes should best be used to normalize qRT-PCR results. The programs geNorm and NormFinder both ranked Xbp1, Tbp, CAPON and Stx16 as the genes with the highest expression stability, albeit in a different order. As briefly mentioned above, the use and comparison of both programs is highly recommended, because in principle, results can be strongly biased by the analytical approach selected [23].

Because the results of geNorm and NormFinder are largely concordant, we conclude that there are no significantly coregulated genes in the seven genes studied. Thus, there is no need to choose genes with opposite regulation in induced and control samples. It is largely accepted that at least three reference genes should be used for normalization, as every additional gene increases the robustness of the NF. Based on the analyses using geNorm and NormFinder, we consider Tbp, CAPON and Xbp1 as a good set of internal reference genes for expression analysis of *Chaoborus*-treated daphnids (Table 5). The results of the NormFinder II analysis (Table 4) showed that the three highest ranked genes (Xbp1, Tbp and CAPON) have a slight tendency towards higher expression in the induced compared to the non-induced samples. It might therefore be advisable to include Stx16 as a fourth reference gene, because of its opposing expression, as advocated by Andersen et al. [21].

**Table 5:** Most stable reference genes and optimum number of reference genes calculated by geNorm and NormFinder

	**geNorm**	**NormFinder**	
		**1**^**st **^**analysis**	**2**^**nd **^**analysis**
	
	**Tbp/CAPON**	**Xbp1**	**Xbp1**
	**Xbp1**	**Tbp**	Tbp
	
	Stx16	CAPON	CAPON
	
	GAPDH	Stx16	**Stx16**
	
	aTub	aTub	
	
	MMP	GAPDH	
	
		MMP	
	
Optimum number	3	2	2

Genes are ranked according to Figure 1A (geNorm) and to their stability values in Table 3 and 4, respectively (NormFinder). In bold letters: The recommended optimum combination of genes.

The genes Xbp1 and CAPON have not yet been used as normalization genes in any study. However, the systematic validation in this study provides evidence that they are suitable reference genes under these experimental conditions despite comparatively low transcription levels (CP > 25). Most importantly, they are much more stably expressed between experimental groups than the classical housekeeping genes aTub and GAPDH. In general, variation is expected to be inversely proportional to the amplified target amount but low variation despite high CP (Ct) values has been observed in other studies as well [e.g. [24]].

### Evaluation of the selected reference genes

**Figure 2:**
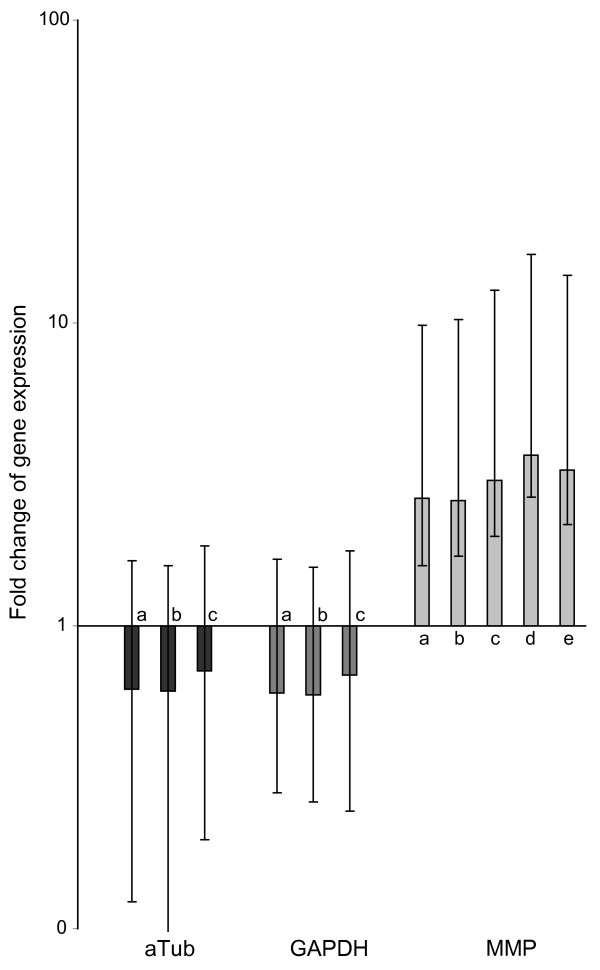
**Differential expression of aTub, GAPDH and MMP evaluated with REST using different normalization strategies.** Program and genes used as reference genes for normalization: a) geNorm: Tbp/Xbp1/CAPON; b) NormFinder I: Tbp/Xbp1; c) NormFinder II: Xbp1/Stx16; d) the classical house-keeping genes Tbp/aTub/GAPDH; e) all genes, i.e.: Tbp/aTub/GAPDH/Stx16/Xbp1/CAPON. Boxes represent the interquartile range, or the middle 50% of observations. The dotted line represents the median gene expression. Whiskers represent the minimum and maximum observations.

The choice of reference genes can have a strong impact on the results in relative expression studies [25]. To test the impact of reference gene selection and to evaluate the suitability of the reference genes selected in this study, the differential expression of MMP between *Chaoborus*-induced and non-induced daphnids was assessed using three different sets of reference genes - (1) the most stable genes identified by geNorm and NormFinder, (2) the classical housekeeping genes (aTub, GAPDH and Tbp) and (3) all six candidate reference genes (Table 6, Figure 2). Furthermore, we tested aTub and GAPDH, which were deemed unstable with high intergroup variation, to determine if this was due to unspecific fluctuations or to a significant differential expression of both genes (Table 6, Figure 2). In addition, we assessed the effects of using single traditional housekeeping genes (aTub and GAPDH) for normalization of expression levels only (additional file 1).

**Table 6:** Results of the gene expression analysis with REST for aTub, GAPDH and MMP with different normalization strategies

Normalized against	Target gene	x-fold change in gene expression*	SE range	P
geNorm: Tbp, Xbp1, Capon	aTub	0.62	0.50 - 1.02	0.009

	GAPDH	0.60	0.32 - 1.06	0.007

	MMP	2.64	1.06 - 7.18	0.003

NormFinder I: Tbp, Xbp1	aTub	0.61	0.51 - 0.97	0.001

	GAPDH	0.59	0.33 - 0.97	0.002

	MMP	2.60	0.90 - 7.68	0.005

NormFinder II: Xbp1, Stx16	aTub	0.71	0.51 - 1.13	0.034

	GAPDH	0.69	0.44 - 1.08	0.013

	MMP	3.02	1.05 - 9.80	0.003

Classical HKGs: Tbp, aTub, GAPDH	MMP	3.66	1.00 - 13.16	0.003

All genes	MMP	3.27	1.11 - 11.07	0.002

*x-fold change in gene expression in the induced samples compared to the control group.

Expression analyses were done using the relative expression software tool REST v. 2.0.7 [26]. REST applies the efficiency-corrected comparative CP method [27] and performs randomization tests to estimate a sample's expression ratio and the likelihood of up or down regulation, taking into account several reference genes and the individual amplification efficiency of each gene. The P values obtained from the randomization tests in REST indicate the likelihood of observing differences between sample and control groups due to chance alone. They were calculated by 10,000 random reallocations of samples and controls between the groups and counting the number of times the relative expression on the randomly assigned group is greater than the sample data (see REST manual http://www.gmo-qpcr-analysis.com/REST2008_Manual_v207.pdf).

Differential expression measurements of MMP indicated a significant upregulation irrespective of the normalization strategy (Table 6, Figure 2). Similarly, both traditional 'housekeeping' genes aTub and GAPDH were reported significantly downregulated in induced animals, which supports the finding of a significant downregulation on protein level for aTub [28] and differs from the results of Schwarzenberger et al. [10] for *D. magna*.

Differential expression levels reported by the REST program depended on the reference genes selected for normalization (Figure 2). For MMP they ranged from 2.64 to 3.66 in the induced samples, whereas for aTub (0.61 to 0.71) and GAPDH (0.59 to 0.69) fluctuations were less prominent. Since REST uses expression ratios rather than CP raw values for randomization tests and the data visualized in the Whisker box plots (Figure 2, additional file 1) often portray lopsided distributions with standard errors.

Because aTub and GAPDH show variations in expression, they are not suitable reference genes for such experiments with *D. pulex*. Even though no strong difference on the regulation of MMP is observed the impact reference genes have on the results become evident when using classical housekeeping genes aTub and GAPDH as reference genes. When selecting aTub as a single reference gene for normalization, REST reports a significant upregulation not only for MMP but also for Xbp1 and Tbp (1.66 and 1.61; see additional file 1a). No differential regulation is then reported for GAPDH due to similar (low) expression. These results highlight the importance of testing for expression stability of reference genes. When selecting GAPDH as the only reference (additional file 1b), MMP is the only gene with significant differential expression, aTub is reported to underlie no significant differential expression. Altogether, the false positive report of differential expression for Xbp1 and Tbp and the false negative report of differential expression for GAPDH when using aTub as reference gene for expression normalization highlights the importance of careful reference gene selection according to the approach outlined in this study.

### Alpha tubulin paralogs in *Daphnia pulex*

Of clear importance for gene expression studies using *D. pulex *is the large number of duplicated gene families, many comprised of recently derived paralogs. In the genome of *D. pulex*, at least five aTub genes were identified (Dappu-318433, Dappu-306726, Dappu-301837, Dappu-315806 and Dappu-315805) http://wfleabase.org. Interestingly, in microarray experiments, members of this gene family have opposing directionality in their expression. This has been observed also for other genes in other taxon groups [e.g. [29]]. This issue might bias the results of the qRT-PCR when primers interrogate more than one aTub gene. In any case, such qRT-PCR markers are avoidable by querying the genome sequence.

### Functional role of MMP

Our finding that MMP is significantly upregulated by qRT-PCR was expected; this gene is noted in a microarray study (DGC manuscript in preparation) to be upregulated in juvenile daphnids exposed to *Chaoborus *kairomone. According to the most recent JGI annotation, the MMP gene found is a Meprin A metalloprotease. NCBI BLAST matches it to genes that are members of the protein family Astacin (peptidase family M12A; PF01400). MMPs are known to play an important role in development, in particular in degrading and processing proteins, and are associated with cell-cell signalling pathways [17-19]. Although our data clearly identified MMP as a differentially expressed candidate gene, subsequent analyses should now focus on analyzing the function of this gene product in *D. pulex *to understand its relevance in the context of predator-induced defences.

## Conclusions

Our results suggest that Xbp1, Tbp, CAPON and Stx16 are suitable internal reference genes for studying relative gene expression levels in *D. pulex *that are challenged by *Chaoborus *predation. Two traditional housekeeping genes, GAPDH and aTub, were studied with qRT-PCR and found to have a strong expression variation and were significantly downregulated. One candidate gene with assumed differential expression, MMP, was found to be significantly upregulated. Using aTub as reference gene leads to a strong bias in reported expression levels emphasizing the importance of thorough reference gene evaluation prior to target gene expression profiling. In particular, for further studies investigating transcriptional responses of *Daphnia *to other treatments we recommend to experimentally verify stable expression of reference genes prior to data acquisition in order to improve accuracy and reliability of qRT-PCR data.

## Methods

### Test species

*Daphnia pulex *(Clone R9) were used for our study. *Daphnia *medium consisted of charcoal filtered tab water. All *Daphnia *cultures and experiments were conducted at 20°C with a 16/8-h light/dark cycle. Culture daphnids were maintained at a density of ~50 animals per litre and fed daily with the unicellular green algae *Scenedesmus *spp. which was cultured in the laboratory.

### Predator assay

Induction of *D. pulex *was carried out by incubating age- synchronized adult females in 1 l glass beakers with a nylon net cage containing 20 *Chaoborus *sp. larvae so that the adult females and their offspring had contact with *Chaoborus *kairomones but would not be preyed upon. *Chaoborus *larvae were fed daily with 40 juvenile daphnids. The control group was reared in similar glass beakers with nylon net cages that did not contain *Chaoborus *larvae.

Twelve biological replicates of induction and control, respectively, were conducted.

Neonate daphnids were separated daily from the mothers. They were reared under the same conditions until they reached the second juvenile instar, and batches of 50 animals were preserved in 20 μl RNA*later *(Qiagen) and stored at 4°C until RNA extraction. A representative amount of induced and non-induced juveniles were checked for the presence and absence of neckteeth, respectively. Batches that did not show the appropriate phenotypic expression were discarded.

### RNA isolation and cDNA synthesis

RNA*later *was decanted from the specimens and RNA was extracted using the MasterPure Complete DNA and RNA Purification Kit (Epicentre) according to the manufacturer's protocol. The purified nucleic acids were resuspended in 30 μl of RNAse free water. The integrity of the RNA samples was checked with Experion RNA StdSens Analysis Kit (Bio-Rad) and concentration and purity with the NanoDrop ND-1000 spectrophotometer (NanoDrop Technologies). An amount of 1 μg of each extraction was reverse-transcribed with QuantiTect Reverse Transcription Kit (Qiagen) according to the manufacturer's protocol, which included a 20 min DNAse I digestion prior to reverse transcription. For reverse transcription oligo(dT) primers (1 μM) were used. The cDNA was diluted ten-fold with RNAse free water.

Samples were checked with PCR (GAPDH primer pair) for genomic DNA contamination after the DNAse I digestion. An additional positive control was the single product of 80 bp in the qRT-PCR reactions with the MMP primer pair which was designed to span an intron.

### Identification of candidate reference genes

The sequences of putative genes with microarray and EST support and a gene prediction (data available from http://wfleabase.org) were aligned using tBLASTx against NCBI (National Center for Biotechnology Information) nucleotide database sequences to find homologue genes and assign a putative function. Unpublished microarray data (manuscript in preparation) were then taken as basis to estimate expression stability.

### Design and validation of qRT-PCR primers

For primer design, the Primer3 v. 0.4.0 software [30] was used with the following settings differing from the default parameters:

Primer size of 20-27 bp, amplicon size 130-150 bp, melting temperature 60-61°C; maximum temperature difference of 0.5°C, maximum length of a polynucleotide repeat 3, and a number of consecutive Gs and Cs at the 3' end of 1.

Primers were designed preferentially for the 3'-exon. The primers for MMP span the last 3'-intron. To check for mispriming primers were blasted (BLASTn) against the genome of *D. pulex*. Primers with a binding energy ΔG of less than -3 kcal/mole and -2 kcal/mole for internal hairpins and hairpins at the 3'-end, respectively, as well as those with less than -6 kcal/mole and -5 kcal/mole for internal or 3' self and cross dimers, respectively, calculated with Beacon Designer Free Edition of Premier Biosoft International, were excluded.

### Real-time quantitative PCR

The PCR mix consisted of 2 μl cDNA (equivalent to approximately 10 ng cDNA), 10 μl of the DyNAmo Flash SYBR Green qPCR Kit (Finnzymes), primer concentrations of 300 nM of forward and reverse primers each (Stx16, Tbp, MMP, CAPON), 100 nM each (aTub, GAPDH), 400 nM each (Xbp), and PCR-grade water up to a total volume of 20 μl.

Reactions were performed in triplicates and a no-template control was included. Every gene was tested for all biological replicates on a separate 96-well plate.

PCR reactions were performed using the DNA Engine Opticon 2 Two-Color Real-Time PCR Detection System (Bio-Rad) and the following conditions: 10 min at 95°C and 40 cycles of 95°C for 15 s, followed by 60°C for 1 min; finally 1 min at 55°C. Amplification specificity was verified based on the melting curve which was obtained by heating in steps of 0.3°C from 60°C to 95°C.

### Analysis of candidate reference genes

Optical raw data (not baseline corrected) were exported from the Opticon Monitor software v. 3.1 (Bio-Rad) into MS Excel (Microsoft) and processed with the program LinRegPCR v. 11.0 [31,32]. LinRegPCR determines CP values for each reaction and a mean PCR efficiency corresponding to a primer pair by a linear regression fit to the data in the exponential phase of a reaction.

The BestKeeper descriptive statistical method was applied [9] on the CP values determined by LinRegPCR.

For subsequent analysis with the programs geNorm v. 3.5 [7] and NormFinder v. 0.953 [21], CP values were converted to linear values.

### Gene expression analysis and reference gene evaluation

Gene expression analysis of MMP in the induced versus the non-induced samples was carried out with the relative expression software tool REST v. 2.0.7 [26] REST analyzes gene expression data (based on CP values) with particular emphasis on describing and visualizing uncertainty in expression ratios by introducing a randomization test, calculating confidence intervals and standard errors [see [26]]. Whisker box plots provide a visual representation of variation for each gene.

In this study CP values and mean efficiencies determined with LinRegPCR were used for analysis and randomization tests were performed with 10,000 iterations to assess the significance. The MMP expression levels were normalized using five different strategies. Furthermore, gene expression analysis was carried out for Tbp, Stx16, Xbp1, CAPON and MMP using the classical 'housekeeping genes aTub and GAPDH as reference.

## Abbreviations

*Genes: *aTub: alpha tubulin; GAPDH: glyceraldehyde-3-phosphate dehydrogenase; MMP: matrix metalloproteinase; Stx16: syntaxin 16; sucDH: succinate dehydrogenase; Tbp: TATA binding protein; UBC: ubiquitin conjugating enzyme; Xbp1: X-box binding protein 1.

*Other: *BLAST: basic local alignment search tool; CP: crossing point; NCBI: National Center for Biotechnology Information; NF: normalization factor; SD: standard deviation; qRT-PCR: quantitative real-time reverse transcription polymerase chain reaction.

## Authors' contributions

KIS, FL and RT designed the research. KS and FL carried out the molecular genetic and statistic analyses, made the data interpretation and drafted the manuscript. CM helped with the data interpretation and provided bioinformatic support. MEP, DG and JKC provided the microarray data. RT, MEP, DG and JKC revised critically the manuscript. RT provided financial support to the study. All authors read and approved the final manuscript.

## Supplementary Material

Additional file 1 **Results of the gene expression analysis with REST when using aTub (1a) and GAPDH (1b) as reference genes for expression normalization**. Click here for file
