# Supplementary material for: Predator-induced defences in Daphnia pulex: Selection and evaluation of internal reference genes for gene expression studies with real-time PCR
Source: BMC Mol Biol. 2010 Jun 29;11:50. doi: 10.1186/1471-2199-11-50 (PMC3148505; doi:10.1186/1471-2199-11-50)
Supplement: Additional file 1 — Results of the gene expression analysis with REST when using aTub (1a) and GAPDH (1b) as reference genes for expression normalization. [file 1471-2199-11-50-S1.pdf]

Additional file 1: Results of the gene expression analysis with REST when using aTub (1a) and GAPDH (1b) as reference genes for expression normalization.

Table 1a: Results of the REST analysis using aTub as reference gene. Three genes appear significantly upregulated according to this analysis revealing the strong bias the selection of an unsuitable reference gene can have.

Gene	Type	Reaction Efficiency	Expression	Standard Error	95% C.I.	P-Value	Result
aTub	Reference	0.91	1.00				
Xbp1	Target	0.93	1.66	0.96 - 2.43	0.69 - 12.30	**0.006**	upregulated
Stx16	Target	0.93	1.19	0.80 - 1.62	0.53 - 4.41	0.279	
Tbp	Target	0.9	1.60	0.98 - 2.02	0.81 - 17.64	**0.002**	upregulated
G3PDH	Target	0.93	1.39	0.73 - 2.69	0.58 - 16.36	0.275	
MMP	Target	0.93	4.31	0.94 - 15.79	0.29 - 119.80	**0.002**	upregulated
CAPON	Target	0.94	1.57	0.787 - 2.15	0.54 - 40.15	0.097	

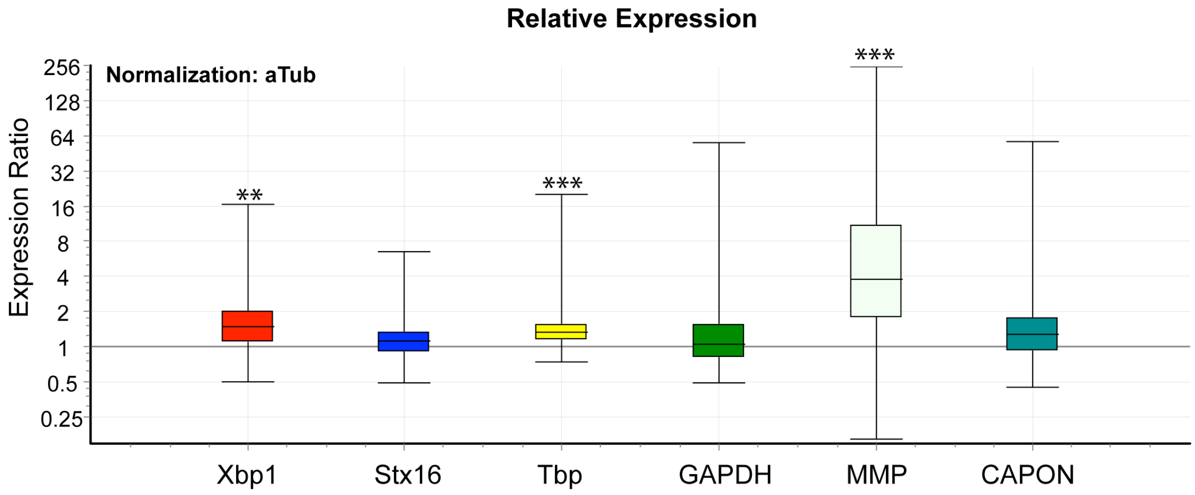

Figure 1a: Boxplot of the results of the REST analysis using aTub as reference gene. Boxes represent the interquartile range, or the middle 50% of observations. The dotted line represents the median gene expression. Whiskers represent the minimum and maximum observations. Significance: *=P<0.001, ***=P<0.005.

Electronic supplement 1b:

Table 1b: Results of the REST analysis using GAPDH as reference gene. Only MMP is reported with significant differential regulation.

Gene	Type	Reaction Efficiency	Expression	Standard Error	95% C.I.	P-Value	Result
G3PDH	Reference	0.93	1.00				
Xbp1	Target	0.93	1.20	0.69 - 2.45	0.14 - 3.72	0.495	
Stx16	Target	0.93	0.86	0.65 - 1.39	0.11 - 1.88	0.606	
Tbp	Target	0.9	1.15	0.76 - 2.31	0.09 - 4.31	0.639	
aTub	Target	0.91	0.72	0.37 - 1.36	0.06 - 1.72	0.273	
MMP	Target	0.93	3.10	0.68 - 13.81	0.19 - 39.97	**0.013**	upregulated
CAPON	Target	0.94	1.13	0.59 - 2.27	0.13 - 10.47	0.662	

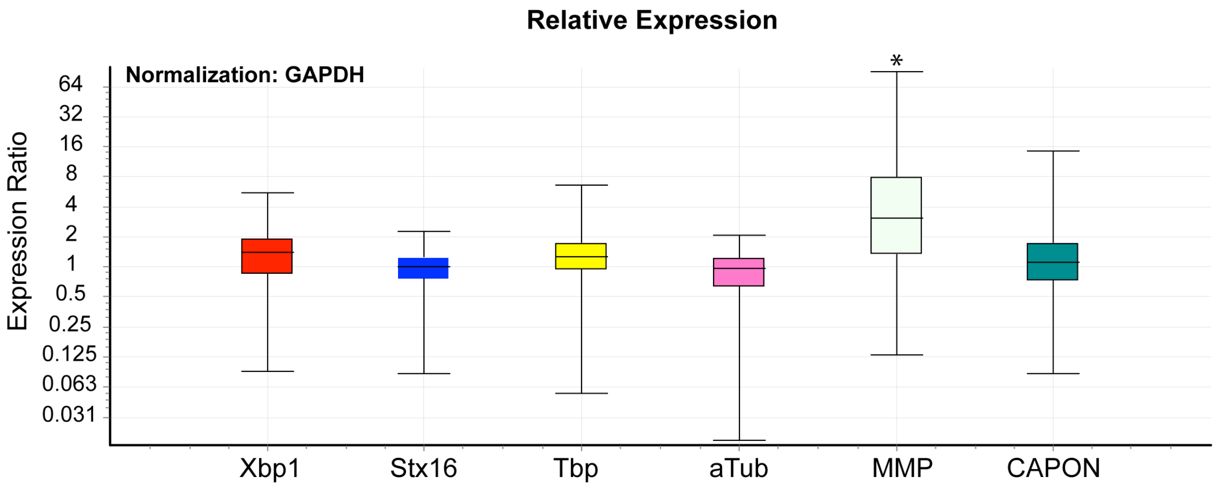

Figure 1b: Boxplot of the results of the REST analysis using GAPDH as reference gene. Boxes represent the interquartile range, or the middle 50% of observations. The dotted line represents the median gene expression. Whiskers represent the minimum and maximum observations. Significance: *=P<0.05.
